# Late Diagnosis of Bilateral Choanal Atresia: A Case Report of a Nine-Year-Old Girl

**DOI:** 10.7759/cureus.27203

**Published:** 2022-07-24

**Authors:** Winga Foma, Haréfétéguéna Bissa, Saliou Adam, Essobozou P Pegbessou, Bathokedeou Amana

**Affiliations:** 1 Department of ENT/Head and Neck Surgery, University of Lomé, Lomé, TGO; 2 Department of Stomatology and Maxillofacial Surgery, University of Lomé, Lomé, TGO

**Keywords:** transpalatal choanoplasty, late diagnosis, nasal obstruction, congenital, choanal atresia

## Abstract

Bilateral choanal atresia is a surgical emergency because of the risk of neonate death from acute asphyxia if treatment is delayed. Its diagnostic confirmation is often endoscopic or CT scan and requires a search for associated malformations.

We present the case of a nine-year-old girl who was referred to the ENT department with suspected adenoid pathology. Her medical history showed respiratory distress at birth treated as a neonatal infection. We suspected bilateral choanal atresia due to the absence of fogging on mirror test and failure to pass a 6Fr or 8Fr suction catheter through the nasal cavity into the nasopharynx. Facial CT confirmed the presence of bilateral mixed osteo-membranous choanal atresia. Transpalatal choanoplasty was successfully performed with pre and postoperative endoscopic examination.

This clinical case adds to the limited literature on bilateral choanal atresia diagnosed long after birth, raising once again the lack of knowledge of choanal atresia by some health workers, emergency neonatal care, the mechanism of breathing in the newborn, and the management of this malformation. Transpalatal choanoplasty is a good alternative when technical conditions do not allow an endoscopic endonasal approach.

## Introduction

Choanal atresia (CA) is a rare birth defect characterized by imperforation of the posterior part of one or both nasal cavities. Neonates are obligate nasal breathers making bilateral CA a surgical emergency requiring prompt management for airway control. Its diagnosis is clinical, sometimes requiring endoscopic or CT confirmation and a search for associated defects. The treatment of CA is surgical and can be performed either as an endonasal or endobuccal procedure. Several surgical approaches have been described (transnasal, transpalatal, sublabial-transnasal, transantral, and transseptal) but transnasal and tranpalatal are commonly used [1]. Recurrence is possible. For example, Attya et al. reported in their study that the average total number of operations patients needed was 3.43 [2]. The discovery of bilateral CA distant from the perinatal period is rarely reported in scientific works. We report the first described case of late diagnosis of bilateral CA in Togo in a nine-year-old girl and discuss our workup and treatment.

## Case presentation

**Figure 1:**
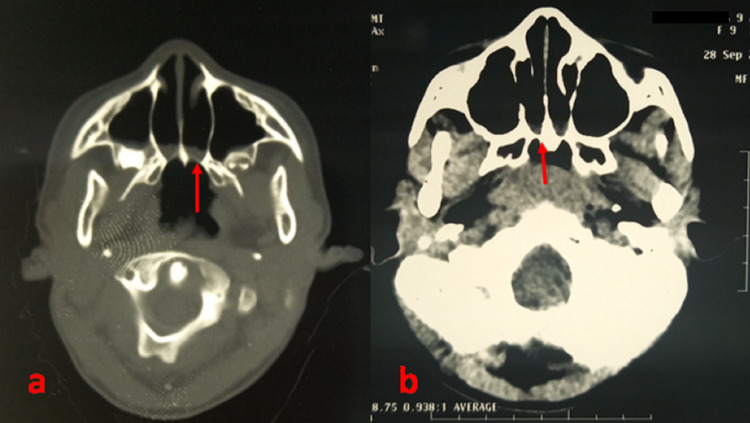
CT scan of an osteomembranous choanal atresia (red arrow) in axial sections bone window (a) and parenchymal window (b).

A nine-year-old girl was referred to our department for a suspected adenoid pathology. Clinical history reported nasal obstruction and chronic bilateral mucosal rhinorrhea since the first weeks after birth. Birthing history revealed that the mother had been followed in a peripheral care unit and no gravidic or non-pregidic pathology was noted. At birth, respiratory distress was reported and was treated as a neonatal infection for a two-week period. No measures to secure the airways had been reported. From then on, the evolution was marked by mouth breathing, breathing difficulties during feeding, restless sleep with apnea episodes, and delayed growth in height and weight. The ENT physical examination noted a weight of 21 kg, mouth breathing, no fogging on the mirror test, and a positive rubber catheter test. Bilateral choanal atresia was strongly suspected. Facial CT confirmed bilateral mixed osteomembranous choanal atresia (Figure 1).

**Figure 2:**
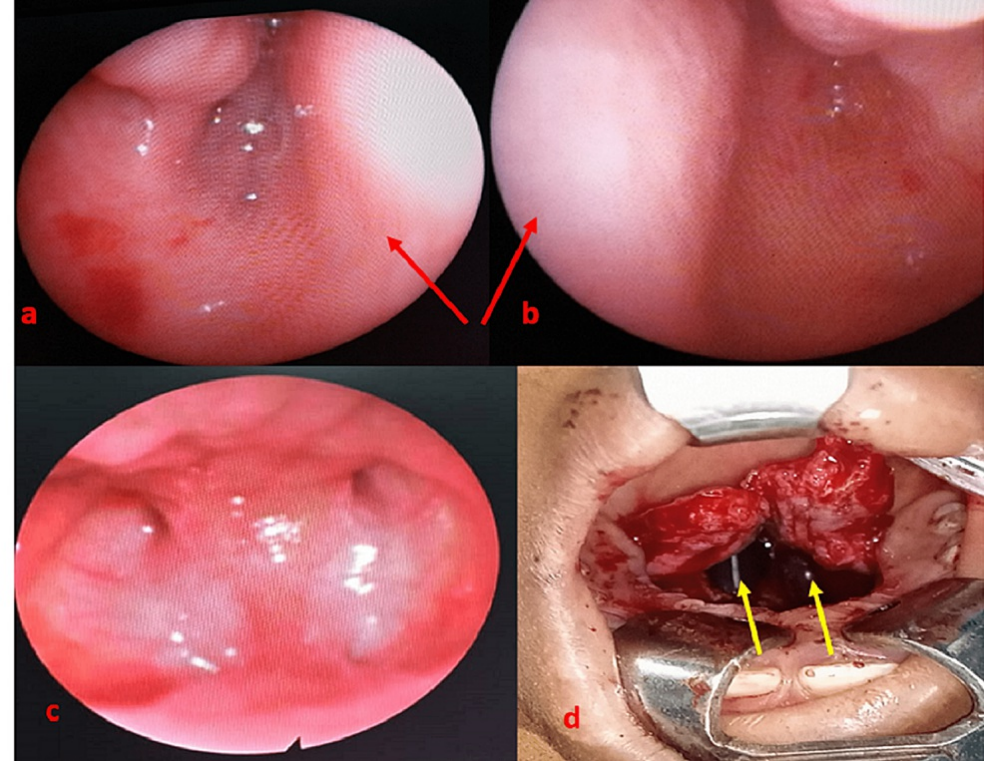
Endoscopic aspects of choanal atresia on the right (a), on the left (b) with the nasal septum indicated by the red arrow, in posterior view (c), and in intraoperative view (d) showing stents (yellow arrows).

There was no other associated defect that could define a syndrome. The preoperative biological workup was normal. Under general anesthesia, after endoscopic observation of the atretic region (Figures 2a, 2b, 2c), the patient underwent a transpalatal osteotome choanal divulsion (Figure 2d).

**Figure 3:**
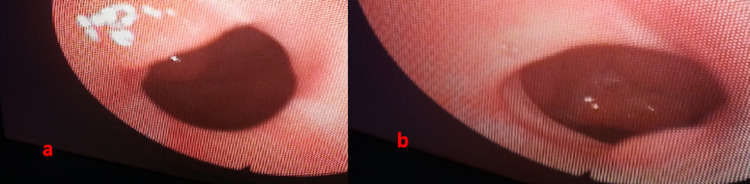
Endoscopic aspects of the choana on the right (a) and left (b).

Nasal cavity stents was maintained in place for three months with local saline instillations. At six months postoperatively, with the parental assistance in rehabilitation, nasal breathing was effective. At one year, the choanae were well patched (Figure 3) and the patient had improving weight gain.

## Discussion

Our observation adds to the 10 cases of bilateral CA diagnosed long after birth, already reported in the literature [3,4], raising once again the lack of knowledge of choanal atresia by some health workers, emergency neonatal care, the mechanism of breathing in newborn, and the management of this malformation.

To provide better care for CA in newborns, it is critical to segregate the different possibilities of nasal obstruction. At birth, nasal obstruction may be related to neonatal rhinitis, rhino-septal deviations due to obstetric trauma, nasal dysgenesis (stenosis of the piriformis orifice anteriorly, stenosis of the nasal aperture in the middle part, and CA posteriorly), cerebral malposition (meningoceles and meningoencephaloceles), congenital endonasal masses (nasolacrimal cysts, dermoid cysts, nasal hemangiomas), embryonic tumors, or arhinia [5,6]. With an estimated incidence of 1/5000 and 1/7000 births, CA is more rarely bilateral and may sometimes be part of a polymalformative framework, the most frequent of which is CHARGE (colobomas, cardiac (heart) defects, choanal atresia, retardation of growth and cognition, genitourinary anomalies, and ear anomalies) syndrome [1,4,6-8]. Diagnosis should be made immediately after delivery by passing a small rubber catheter through each newborn's nasal cavity to check for patency. This catheter test should be done routinely in the delivery room given the severity of undiagnosed bilateral CA. The present observation also shows the too frequent tendency to mention only tonsillar pathology before breathing difficulties during the sleep of the child, sometimes leading to long medical work. A careful examination taking into account the perinatal period and a simple methodical examination would be of great help for the diagnosis of nasal obstructions, especially in places where endoscopic and radiological explorations are not easily accessible.

Due to the high position of the larynx in newborns, breathing through the mouth is almost impossible. Breathing through the mouth is obstructed due to the fact that the epiglottis is very close to the soft palate and that tongue is in close contact with both the soft and hard palate in the neonatal period [3]. In bilateral CA, respiratory distress with cyanosis is very quickly observed, improving with crying and worsening with suckling. It is therefore rare to find cases of bilateral CA one week after birth because of its incompatibility with life [4]. As discussed by other researchers, this observation further raises the question of whether all newborns are mandatory nasal respirators.

The care of bilateral CA requires emergency medical procedures such as placement of an adapted Guedel’s cannula or orotracheal intubation, diagnostic confirmation by nasal endoscopy and/or craniofacial CT scan, and then prompt surgical care. In our resource-limited settings, direct endoscopic exploration under general anesthesia upon strong clinical suspicion could help avoid CT scans and reduce the cost of treatment, which is often borne by patients.

Surgically, the endonasal and transpalatal approaches are the most frequently used, but the nasal endoscopic approach is increasingly preferred in choanoplasty. Because of the lack of all the endoscopic surgical equipment adapted to our patient who was nine years old and 21 kg, the transpalatal approach was used successfully with pre and postoperative endoscopic controls. Restenosis, the most frequent complication of CA cure, is possible whatever the surgical technique used. Nasal cavity stenting does not seem to avoid the risk of restenosis according to some researchers and its impact on the surgical outcome is still a matter of controversy [2,3,9]. Adjuvant therapy such as local application of mitomycin-C or corticosteroid eluting stent placement to avoid restenosis have been reported [2,10,11].

## Conclusions

Bilateral CA is a rare and serious defect due to the risk of neonatal asphyxia. Adherence to a newborn care protocol, including systematic verification of nasal cavity patency, could help prevent at worst some neonatal deaths and late diagnosis of CA at best. Transpalatal choanoplasty appears to be a good alternative when technical conditions do not allow an endonasal endoscopic approach.
